# Construction of the preoperative staging prediction model for cervical cancer based on deep learning and MRI: a retrospective study

**DOI:** 10.3389/fonc.2025.1557486

**Published:** 2025-04-02

**Authors:** Xuhao Dai, Xiaoxian Ye, Jiangping Ren, Jiming Yang, Yingying Zhou, Zhaoyang Ma, Pengrong Lou

**Affiliations:** Department of Radiotherapy and Chemotherapy, The First Affiliated Hospital of Ningbo University, Ningbo, China

**Keywords:** cervical cancer, preoperative staging, deep learning, HRNe, MRI

## Abstract

**Background:**

Cervical cancer remains a significant global health concern, particularly for women. Accurate preoperative staging is crucial for treatment planning and long-term prognosis. Traditional staging methods rely on manual imaging analysis, which is subjective and time-consuming. Deep learning-based automated staging models offer a promising approach to enhance both accuracy and efficiency.

**Methods:**

This study retrospectively analyzed preoperative MRI scans (T1 and T2 stages) from 112 cervical cancer patients. Seven deep learning models—DenseNet, FBNet, HRNet, RegNet, ResNet50, ShuffleNet, and ViT—were trained and validated using standardized preprocessing, data augmentation, and manual annotation techniques. Convolutional neural networks were employed to extract multidimensional imaging features, forming the basis of an automated staging prediction model.

**Results:**

Among all tested models, HRNet demonstrated the best performance, achieving an accuracy of 69.70%, recall of 68.89%, F1-score of 68.98%, and precision of 69.62%. ShuffleNet ranked second, with slightly lower performance, while ViT exhibited the weakest predictive ability. The ROC curve analysis confirmed HRNet’s superior classification capability, with an AUC of 0.7778, highlighting its effectiveness in small-sample datasets.

**Conclusion:**

This study confirms that deep learning models utilizing MRI images can enable automated cervical cancer staging with improved accuracy and efficiency. HRNet, in particular, demonstrates strong potential as a clinical decision-support tool, contributing to the advancement of precision medicine and personalized treatment strategies for cervical cancer.

## 1 Introduction

Cervical cancer is one of the leading malignancies in terms of incidence and mortality among women worldwide, particularly in developing countries, where its incidence ranks second among female malignancies, and 85% of cervical cancer-related deaths occur in these countries (1–4). The most common histological type of cervical cancer is squamous cell carcinoma, which accounts for 70%-80% of all cases, while non-squamous types, such as adenocarcinoma (AC), are often associated with poorer prognosis (5, 6). Treatment plans for cervical cancer are individualized based on tumor stage, histological type, tumor size, patient age, and overall health status (7). For early-stage cervical cancer, surgical resection is the preferred treatment; locally advanced cervical cancer is typically treated with concurrent chemoradiotherapy; and for advanced or metastatic cervical cancer, systemic chemotherapy combined with immunotherapy, targeted therapy, and other modalities are used to prolong survival and improve quality of life. Therefore, accurate preoperative assessment of pathological staging and tumor characteristics is crucial for formulating personalized treatment plans and improving prognosis.

In a retrospective study of 78 cervical cancer patients, Toure M et al. (8) found that preoperative staging often underestimates the true extent of the tumor, with a Cohen kappa coefficient of only 18.07%. In contrast, the consistency between intraoperative staging and postoperative pathological staging was higher (Cohen kappa coefficient of 79%). Underestimation of staging preoperatively may lead to missed optimal treatment opportunities, increasing the risk of surgical failure or tumor residue. The accuracy of radiological assessment is influenced by the individual experience and expertise of the radiologist, particularly when the tumor borders are unclear or the imaging findings are atypical, making staging more difficult. Radiological assessment not only requires high-level professional knowledge but also demands sufficient time and resources for precise analysis, which may be difficult to achieve in a busy clinical setting. Additionally, there is a lack of standardized quantitative criteria for radiological staging, and the results often depend on the subjective judgment of the physician, leading to lower accuracy and consistency. Accurate staging of cervical cancer is essential for determining appropriate treatment strategies, as outlined in multiple international guidelines. A recent study by Restaino et al. (2024) compared the recommendations from major scientific societies, including ESGO, NCCN, ASCO, and FIGO, highlighting the variations and consensus in cervical cancer management across different regions (9). These guidelines emphasize the importance of precise preoperative staging to optimize treatment decisions and improve patient outcomes. However, traditional staging methods remain subjective, underscoring the need for automated, standardized approaches. Therefore, to improve the accuracy of preoperative radiological staging in cervical cancer, there is an urgent need to introduce standardized, objective assessment methods, such as radiomics-based automated analysis and AI-assisted diagnostic tools. These technologies have the potential to address the shortcomings of traditional radiological evaluation, providing more accurate staging predictions and optimizing personalized treatment plans.

Radiomics has a long history of application in cervical cancer, encompassing areas such as survival prediction, lymph node metastasis prediction, treatment response evaluation, and staging prediction. Artificial intelligence-based predictive studies related to cervical cancer staging include: MRI radiomics-based nomograms for lymph node metastasis prediction (10, 11); multiparametric 18F-FDG PET/MR radiomics combined with radiomics analysis and machine learning algorithms for predicting N and M stages (12); a deep learning model developed by Dong T et al. (13) for preoperative lymph node metastasis prediction; and a radiomics model based on T2WI and ADC maps constructed by Wu F et al. (14) to differentiate early (stage I-IIa) from advanced (stage IIb-IV) cervical cancer. These studies provide important references for postoperative staging prediction of cervical cancer; however, the models face challenges such as single-model limitations or insufficient accuracy, and they do not account for the therapeutic differences between early-stage IB3 and IIA2 cervical cancer. The pathological staging prediction in this study is based on treatment guidelines, focusing on the necessity of surgery, to avoid ineffective surgeries and related complications resulting from staging uncertainty.

**Figure 1:**
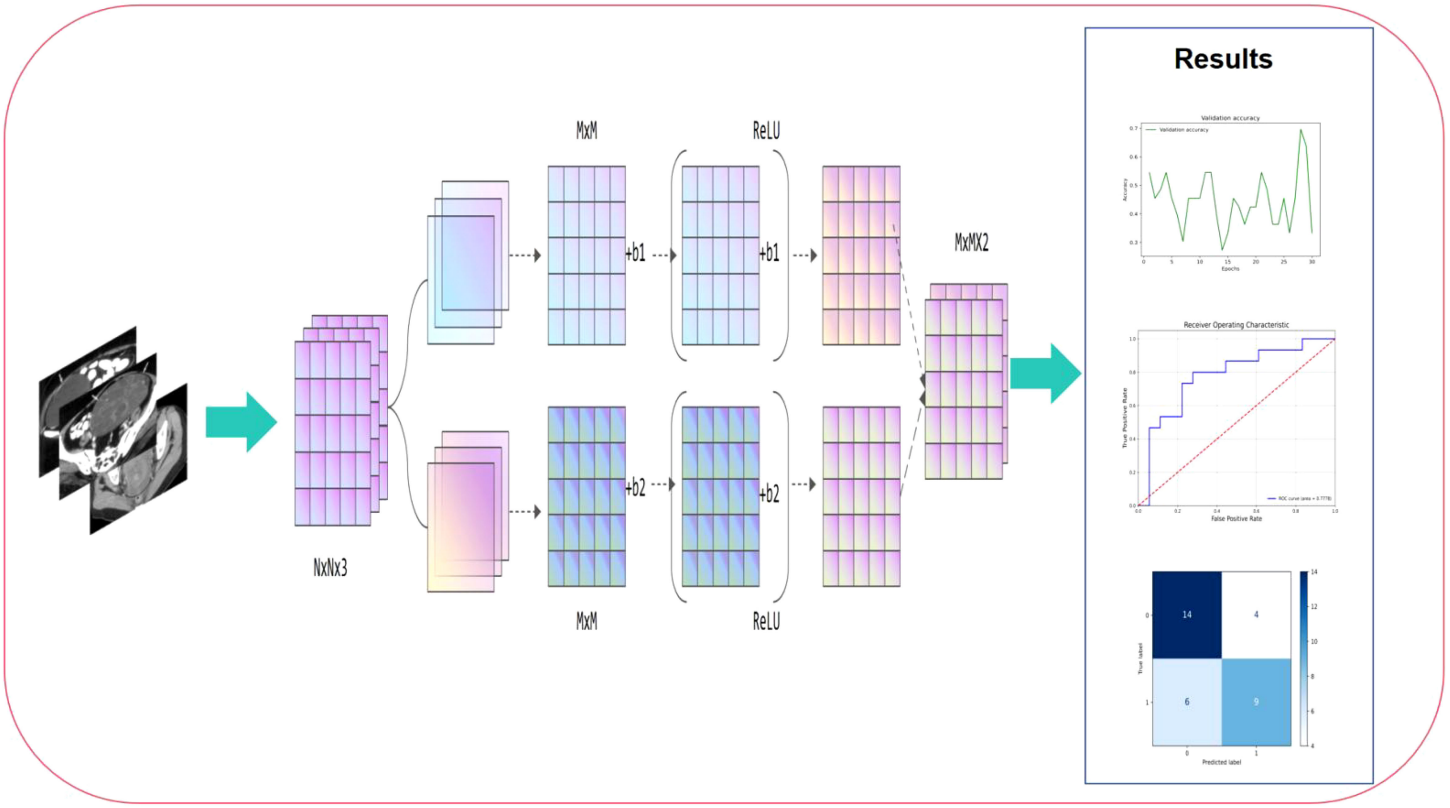
Workflow of this study. The process begins with preoperative MRI image input, which undergoes feature extraction through convolutional layers and non-linear activation functions (ReLU). The extracted high-dimensional features are then processed through multiple computational layers, aggregating critical imaging information for classification. Finally, the right panel presents the model’s performance evaluation, including validation accuracy trends, ROC curve analysis, and confusion matrix, demonstrating its predictive capability.

Based on the MRI data of patients at the T1 and T2 stages, this study used a machine learning fusion method to build a deep learning model that can accurately predict the stage of cervical cancer. During the study, we standardized and enhanced the collected MRI images to improve the quality and efficiency of model training. Subsequently, using high-throughput data feature extraction technology, deep learning methods, especially convolutional neural networks, automatically extracted features in multiple dimensions including texture, shape, density, etc. from MRI images. After training and verification, this study finally developed a deep learning model that can accurately distinguish the stages of cervical cancer, which has the potential to become a new clinical auxiliary diagnostic tool. The workflow of this study is shown in **Figure 1**.

## 2 Method

### 2.1 Study population

This study is a retrospective analysis that included 189 cervical cancer patients who underwent surgical treatment at the First Affiliated Hospital of Ningbo University from June 2019 to June 2024. All cases had clear postoperative FIGO staging, histological subtype, invasion depth, lymph node metastasis status, vascular cancer thrombus, and nerve invasion data. The inclusion criteria were as follows:

1. Age between 18 and 80 years;
2. Histologically confirmed cervical malignancy;
3. MRI examination performed within one month prior to surgery;
4. No neoadjuvant chemotherapy or other treatments before MRI examination.

The exclusion criteria included:

1. Missing key pathological data or incomplete records;
2. Tumor lesions too small to be accurately delineated;
3. Preoperative chemotherapy or treatments that caused changes in tumor imaging;
4. Recurrence or second surgery cases;
5. Unclear images or missing critical scanning sequences.

**Figure 2:**
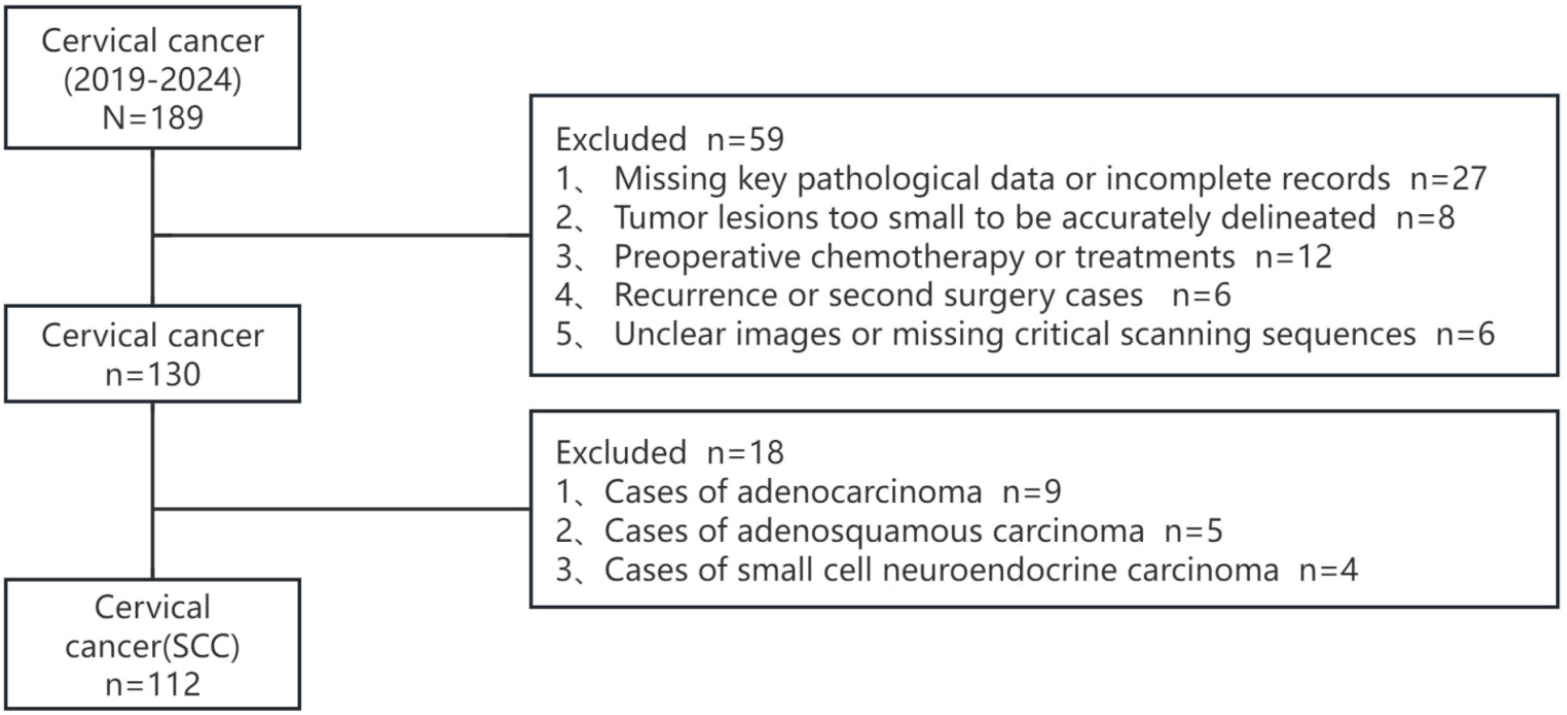
Patient enrollment flowchart.

After screening, 130 patients were initially selected, including 112 cases of squamous cell carcinoma, 9 cases of adenocarcinoma, 5 cases of adenosquamous carcinoma, and 4 cases of small cell neuroendocrine carcinoma. Given the distinct imaging characteristics and treatment responses of different histological subtypes, we focused our analysis exclusively on cervical squamous cell carcinoma (SCC) cases. Cervical adenocarcinoma and adenosquamous carcinoma often present with different tumor morphology and signal intensity on MRI, which could introduce heterogeneity into the model and impact its predictive accuracy. Furthermore, these subtypes may respond differently to treatment, influencing staging decisions and confounding the model’s learning process. By restricting our dataset to SCC, we aimed to enhance model generalizability and ensure more reliable predictions for preoperative staging. To exclude the impact of histological subtype on predictive outcomes, a final cohort of 112 cases of cervical squamous cell carcinoma was included. Patients were randomly allocated into training and validation sets in a 7:3 ratio.The patient enrollment flowchart of this study is shown in **Figure 2**.

According to the 2023 ESGO-ESTRO-ESP guidelines (15), early-stage cervical cancer (IB1, IB2, and IIA1) is recommended for surgical treatment; large cervical tumors (IB3 and IIA2) are recommended for concurrent chemoradiotherapy (CCRT); locally advanced cervical cancer (IIB-IVA) is recommended for concurrent chemoradiotherapy, including external irradiation (pelvic radiation) and internal irradiation; for advanced or widely metastatic cervical cancer (IVB and above), systemic chemotherapy, combined with targeted therapy and immunotherapy, is the primary treatment approach. This study utilized preoperative MRI radiomic features of cervical cancer, along with the treatment strategies recommended by the guidelines, to predict treatment modalities for different stages and conduct a binary classification study between the surgery-recommended group (IA–IB2, IIA1) and the group recommended for concurrent chemoradiotherapy or systemic treatment (IB3, IIA2–IVB). The goal of this study is to provide a reference to avoid unnecessary or missed surgical interventions. All patients signed informed consent. This study was approved by the Ethics Committee of our hospital.

### 2.2 Data preprocessing

In the MRI preprocessing stage, we first performed image culling operations to remove some irrelevant or low-quality areas to ensure the accuracy and representativeness of the data used. Then, we used a variety of denoising techniques, such as Gaussian filtering, to reduce the interference of noise in MRI on model training. In addition, we filtered it to smooth and enhance the edge features of MRI to make it clearer and easier to distinguish. Finally, in order to improve the visibility of key information in MRI, we adjusted the contrast of the image to highlight the tumor area and other clinically relevant features, thereby providing higher quality and more accurate input data for subsequent deep learning model training.

In the process of data augmentation, we used a variety of methods to expand and diversify the training set, including image rotation, translation, flipping, scaling, random cropping, color jittering and other techniques. These operations not only effectively increase the number of samples in the dataset, but also enhance the robustness and generalization ability of the model to various image transformations by simulating different variations in actual scenes. In addition, data augmentation plays an important role in dealing with the problem of class imbalance. By generating more minority class samples, the sample distribution between different classes is balanced, avoiding the model’s bias towards the majority class during training, thereby improving the model’s recognition ability on the minority class.

### 2.3 Manual annotation

To ensure accurate extraction of radiomic features and accuracy of subsequent analysis, all MRI images in this study were manually annotated with regions of interest (ROI). All ROI delineation was performed by two radiologists with more than 10 years of experience, who carefully annotated each image using ITK-SNAP software. The main goal of annotation is to accurately define the tumor area and possible invasion margins to ensure that the extracted features can truly reflect the morphology, size, and biological behavior of the tumor. During the annotation process, the doctors carefully outlined the main body of the tumor and the possible invasion areas around it based on the clarity of the image, the appearance of the lesion, and clinical experience to ensure the comprehensiveness and accuracy of the annotation. To further improve the accuracy and consistency of the annotation, all annotated images were reviewed and adjusted by another radiologist before the final analysis. This review is designed to identify possible errors or inconsistencies and ensure that the annotation of each image is strictly verified, thereby ensuring that the extracted image features are highly representative and clinically relevant.

### 2.4 Construction of stage prediction models

#### 2.4.1 Image feature extraction

Feature extraction is the core of this study. In this stage, we use convolutional neural networks (CNNs) to automatically extract features from two-dimensional MRI images (sections). Through the deep learning model, the network can extract multi-dimensional imaging features from the image, such as texture, shape, edges, etc. These features can accurately reflect the morphology, structure and microenvironment characteristics of the tumor. CNN gradually extracts information at different levels through multiple convolutional layers, thereby capturing the details and global features in the image, ensuring the comprehensiveness and effectiveness of the features. The extracted features are directly input into the deep learning network for training. The model automatically adjusts the network weights through the back-propagation algorithm, and continuously optimizes the representation and weight distribution of the features. This process does not rely on a separate feature screening step, but automatically discovers the most recognizable features through the self-learning ability of the neural network.

#### 2.4.2 Introduction to related models

In recent years, the application of deep learning in medical image analysis has made significant progress, especially in tumor staging, diagnosis and prediction tasks, and various convolutional neural network (CNN) models have been widely used in image classification.

DenseNet (16) improves information flow and gradient propagation through dense connections, alleviating the gradient vanishing problem, but its high number of parameters and computing resource requirements make it have overhead problems when processing large-scale image data. FBNet (17) is a lightweight neural network that optimizes computational efficiency and complexity and is suitable for mobile devices, but its prediction accuracy is not as good as that of large models in complex image analysis. RegNet (18) has a modular architecture and good hardware adaptability, but its lack of flexibility makes it difficult to cope with complex tasks. ResNet50 (19) avoids gradient vanishing through the residual learning mechanism and performs well in tasks such as tumor classification, but its high computational requirements for deep networks limit its real-time application. ShuffleNet (20) greatly reduces the amount of computation by rearranging channels and is suitable for scenarios with limited resources, but its performance in subtle feature recognition is limited. ViT (21) captures the global information of the image by introducing the Transformer architecture and performs well on large-scale datasets, but it has high requirements for data volume and computing resources and is unstable on small datasets.

In order to address the limitations of the above models, we selected HRNet (High-Resolution Network) (22) as the basic model for this study. HRNet excels in medical imaging by preserving high-resolution features and integrating multi-scale information, enhancing tumor staging accuracy. Unlike traditional models, it balances detailed feature extraction and global structure representation, minimizing information loss while maintaining computational efficiency. This makes HRNet particularly suited for analyzing complex medical structures, making it the optimal choice for this study.

#### 2.4.3 Model application details

In this study, we utilized the HRNet (High-Resolution Network) model to enhance the prediction accuracy of preoperative cervical cancer staging. A key advantage of HRNet is its ability to maintain high-resolution feature maps while simultaneously integrating multi-scale information, allowing it to effectively capture both fine-grained local features and global structural details. Given its balance between accuracy and computational efficiency, we selected the HRNet-W32 variant, which features 32-channel widths and a total parameter volume of approximately 27.6 million.

HRNet’s architecture comprises four stages, each containing multiple residual blocks (ResBlock). These blocks incorporate convolutional layers and batch normalization, ensuring stable training and improved feature extraction. The model processes multi-scale information through four parallel resolution paths, progressively merging high-resolution and low-resolution features. This design is particularly effective in handling subtle tumor boundaries and complex texture variations in cervical cancer MRI images, making it well-suited for staging predictions.

To further optimize model performance, we employed stochastic gradient descent (SGD) as the optimizer, setting an initial learning rate of 0.01 with a momentum of 0.9. Additionally, a cosine annealing strategy was implemented to dynamically adjust the learning rate, improving convergence and stability. The model was trained using a batch size of 16 for a total of 100 epochs. To mitigate overfitting, L2 regularization (weight decay coefficient of 1e-4) was incorporated, along with data augmentation techniques, including random cropping, flipping, and brightness adjustment, to enhance generalization and robustness.

### 2.5 Statistical analysis

In this study, we used four commonly used evaluation indicators to measure the performance of the model in the cervical cancer staging task: Accuracy, Precision, Recall and F1-score. Accuracy is used to evaluate the overall classification effect of the model on all samples, and calculate the proportion of correctly classified samples to the total samples (Equation 2). Precision measures the accuracy of the model’s prediction of the positive class, that is, the proportion of all samples predicted to be positive that are actually positive (Equation 1). Recall measures the model’s ability to identify positive samples, indicating the proportion of all samples that are actually positive that are successfully identified as positive by the model (Equation 3). F1-score is the harmonic mean of precision and recall, and is used to evaluate the balance between precision and recall of the model (Equation 4). Through these indicators, we can comprehensively evaluate the classification performance of the model in the cervical cancer staging task, thereby providing a multi-angle evaluation basis for model performance. The following are the relevant formulas:


(1) $$\text{Precision}=\frac{\text{TP}}{\text{TP}+\text{FP}}$$

(2) $$\text{accuracy}=\frac{\text{TP}+\text{TN}}{\text{TP}+\text{TN}+\text{FP}+\text{FN}}$$

(3) $$\text{Recall}=\frac{\text{TP}}{\text{TP}+\text{FN}}$$

(4) $$\text{F}1=2x\frac{\text{Precision}\times \text{Recall}}{\text{Precision}+\text{Recall}}$$

## 3 Results

### 3.1 Characteristics of the patients

**Table 1** displays the baseline characteristics of cervical cancer patients in the training set (n = 77) and test set (n = 33). A total of 110 patients were included in this study for the development and validation of the predictive model, with the dataset randomly divided using the sklearn “Train_test_split” method. In the training set, 44.2% (n = 34) were aged >60 years, while 55.8% (n = 43) were ≤60 years. FIGO stages included IA–IB2 (63.9%, n = 23), IB3 (36.1%, n = 13), IIA1 (60.0%, n = 12), IIA2–IIB (40.0%, n = 8), III (n = 18), IVA (40.0%, n = 2), and IVB (60.0%, n = 3). Positive nerve invasion was observed in 22.1% (n = 17), and vascular tumor embolism was found in 61.0% (n = 47). Stage grouping indicated 41.6% (n = 32) in (IA–IB2) + (IIA1) and 58.4% (n = 45) in IB3 + (IIA2–IVB). In the test set, 42.4% (n = 14) were aged >60 years, and 57.6% (n = 19) were ≤60 years. FIGO stages included IA–IB2 (64.3%, n = 9), IB3 (36.0%, n = 5), IIA1 (26.7%, n = 4), IIA2–IIB (20.0%, n = 3), III (n = 8), IVA (50.0%, n = 1), and IVB (50.0%, n = 1). Positive nerve invasion was observed in 27.3% (n = 9), and vascular tumor embolism was found in 57.6% (n = 19). Stage grouping indicated 48.5% (n = 16) in (IA–IB2) + (IIA1) and 51.5% (n = 17) in IB3 + (IIA2–IVB).No statistically significant differences were observed in clinicopathological characteristics between the training and test sets (P > 0.05).

**Table 1:** Patients baseline characteristics statistics.

Characteristic	Train set(n=77)		Test set(n=33)		P-value
	NO.	%	NO.	%	
Age					
>60	34	44.2	14	45.0	>0.05
<60	43	55.8	19	55.0	
FIGO I					
IA-IB2	25	67.9	10	67.0	>0.05
IB3	12	32.6	5	33.0	
FIGO II					
IIA1	12	60.0	5	62.5	>0.05
IIA2-IIB	8	40.0	3	37.5	
**FIGO III**	18		7		
FIGO IV					
IVA	2	40.0	1	50.0	>0.05
IVB	3	60.0	1	50.0	
Nerve invasion					
Positive	17	22.1	9	27.3	>0.05
Negative	60	77.9	24	72.7	
Vascular Tumor Embolism					
Positive	34	51.5	13	50.0	>0.05
Negative	32	48.5	13	50.0	
Group					
(IA-IB2)+IIA1	21	30.9	9	31.0	>0.05
IB3+(IIA2-IVB)	47	69.1	20	69.0	

### 3.2 Experimental setup

In this study, MRI image data of 112 patients with cervical cancer were divided into training and test sets, with a ratio of 70% (training set) and 30% (test set) to ensure that the model can learn on a sufficient amount of data and perform performance evaluation on independent data. The parameters of all models were optimized by combining grid search and random search, and the ranges of hyperparameters such as learning rate, batch size, and weight decay were determined by pre-experiments. The training of HRNet used the pre-trained version HRNet-W32, whose pre-trained weights were based on the ImageNet dataset. The experiments were conducted on a high-performance computing server equipped with an NVIDIA Tesla A800 GPU (80 GB video memory), an Intel Xeon E5-2698 v4 CPU (2.2 GHz, 20 cores and 40 threads), and running the Ubuntu 20.04 operating system. The deep learning framework used PyTorch 1.12.0, CUDA version 11.6, and cuDNN version 8.4.1. The experimental environment provides sufficient computing resources to ensure efficient training of the model on large-scale MRI image data, while ensuring the stability and accuracy of the training process.

### 3.3 Results of the staging prediction model

#### 3.3.1 HRNet results

**Figure 3:**
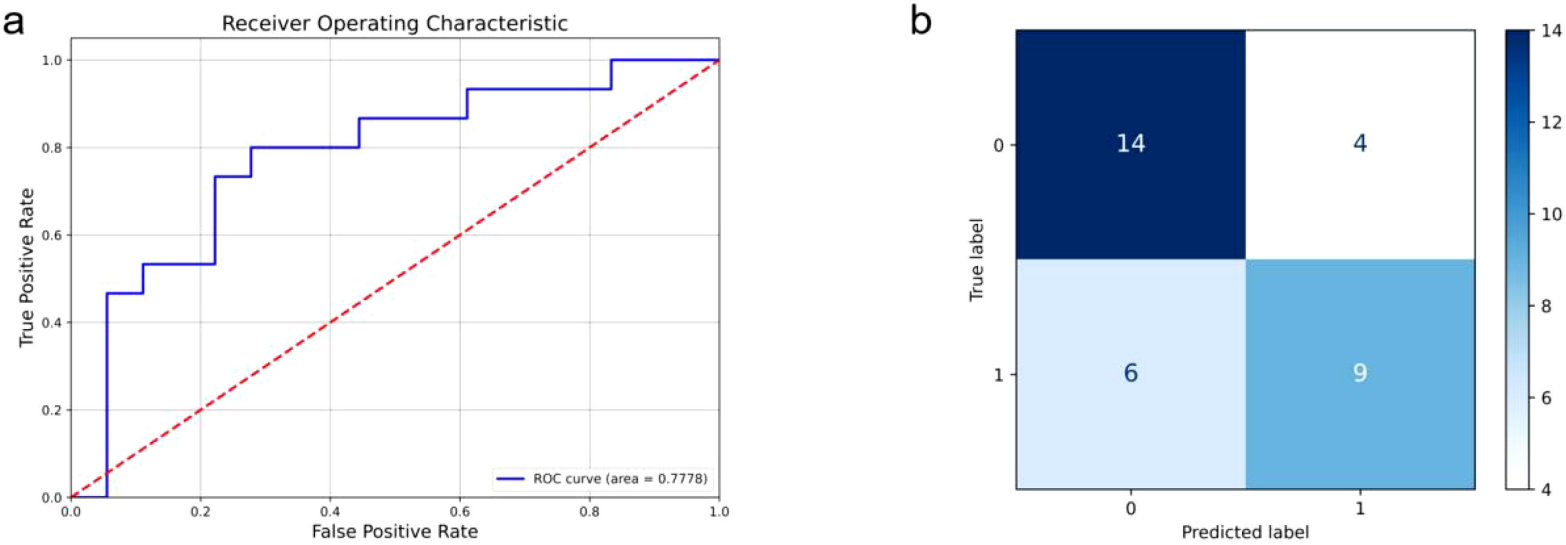
HRNet model results, where **(a)** is the ROC curve and its AUC value, and **(b)** is the confusion matrix.

HRNet performed best in the cervical cancer staging task of this study, with its accuracy (69.70%), precision (69.62%), recall (68.89%) and F1-score (68.98%) all reaching relatively high levels in small samples, showing good predictive performance and balance. The AUC value (0.7778) further proves its strong ability to distinguish between positive and negative samples. This excellent performance is due to HRNet ‘s multi-resolution feature fusion architecture, which can capture global information and detail features while maintaining high-resolution features, especially in tumor boundary recognition and complex texture analysis. The model improves the ability to understand cervical cancer MRI images through layer-by-layer feature extraction and fusion. **Figure 3** shows the ROC curve of HRNet and the corresponding confusion matrix.

#### 3.3.2 Comparison of other model results

**Figure 4:**
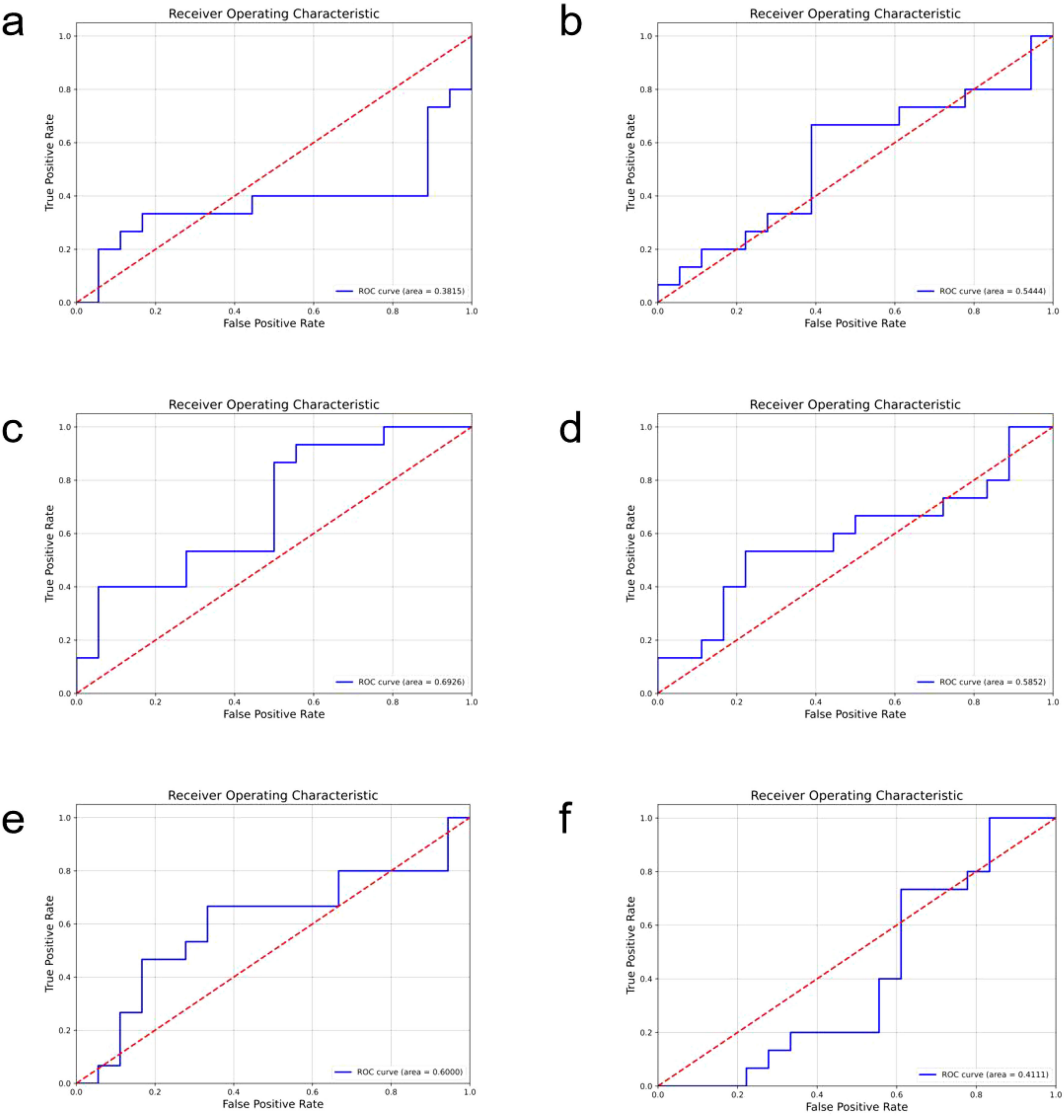
The roc curves of the comparison models are shown, where **(a)** is densenet, **(b)** is FBNet, **(c)** is regnet, **(d)** is resnet50, **(e)** is shufflenet, and **(f)** is the result of vit.

**Figure 5:**
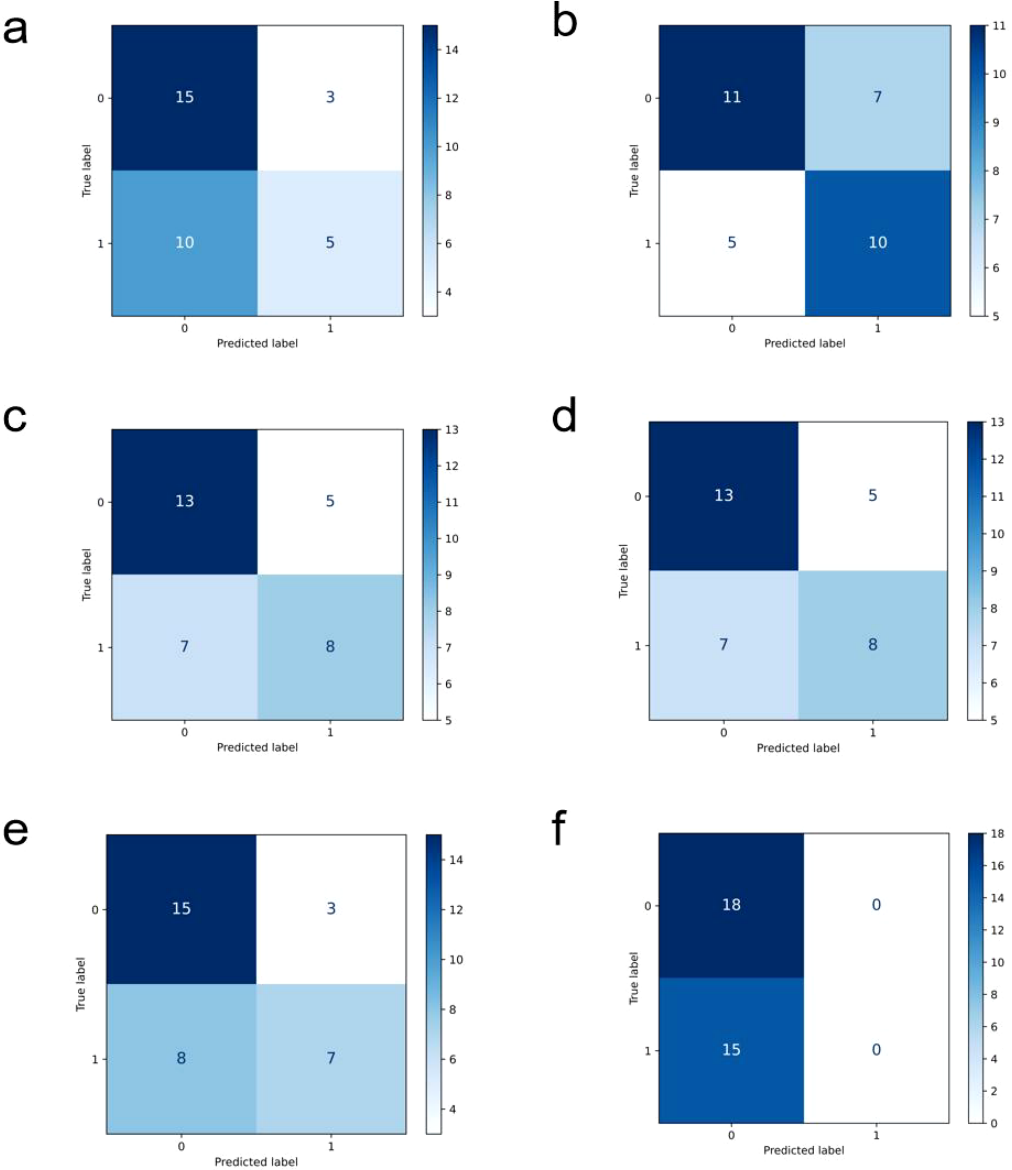
Confusion matrix display of each comparison model, **(a)** is densenet, **(b)** is FBNet, **(c)** is regnet, **(d)** is resnet50, **(e)** is shufflenet, and **(f)** is the result of vit.

In comparison, the performance of other models is relatively inferior (ROC curves of the comparison models are shown in **Figure 4**, confusion matrices are shown in **Figure 5**, and performance comparisons of each model are shown in **Table 2**). FBNet and ShuffleNet showed relatively balanced performance, among which ShuffleNet ‘s accuracy (66.67%) and precision (67.61%) were slightly higher than FBNet, but their AUC values (0.6000 and 0.5444, respectively) were lower than HRNet, indicating their inadequacy in capturing fine-grained features. RegNet and ResNet50 had similar accuracy (both 63.64%) and F1-score (62.78%), but RegNet had a slight advantage in AUC value (0.6926), indicating that it was slightly better in classification discrimination. DenseNet and ViT performed the worst, showing low accuracy (60.61% and 54.55%) and AUC values (0.3815 and 0.4111), respectively. Although DenseNet improves gradient propagation through dense connections, it is not capable of extracting complex image features. ViT, due to its reliance on big data and high resources, performs poorly in small sample scenarios and is difficult to effectively identify local details of tumors. In summary, HRNet ‘s performance exceeds that of other models, proving its superiority and potential in this study.

**Table 2:** Comparison results of various deep learning models.

Model	Acc	Recall	F1-score	Precision
densenet	0.6061	0.5833	0.5662	0.6125
FBNet	0.6364	0.6389	0.6360	0.6379
vit	0.5455	0.5	0.3529	0.2727
regnet	0.6364	0.6278	0.6278	0.6327
resnet50	0.6364	0.6278	0.6278	0.6327
shufflenet	0.6667	0.65	0.6459	0.6761
**HRNet**	**0.6970**	**0.6889**	**0.6898**	**0.6962**

Bold values indicates the best result in all deep learning models.

## 4 Discussion

This study collected and analyzed preoperative MRI data of 112 patients with cervical cancer and constructed an automatic staging prediction model based on deep learning. In the comparison of various deep learning models, HRNet showed the best performance in complex cervical cancer MRI images due to its multi-resolution feature fusion architecture. Specifically, HRNet can capture high-resolution local features and low-resolution global information at the same time, which is particularly important for accurate identification of tumor boundaries and inter-tissue contrast. The experimental results showed that the AUC value of HRNet reached 0.7778, which is better than other models, indicating that it has higher stability and accuracy in distinguishing tumor stages. In addition, by introducing data enhancement and optimizing training strategies (such as cosine annealing learning rate adjustment and regularization), the overfitting problem that may be caused by small sample data is effectively alleviated. The good performance of the model further verifies the applicability of deep learning technology in cervical cancer staging and provides a more efficient automated solution for MRI image analysis.

Our model performs best in the task of preoperative staging of cervical cancer, mainly due to the advantages of the model architecture and its high fit with the task requirements. First, from the perspective of model architecture, HRNet (High-Resolution Network) has unique advantages in processing medical images. HRNet fuses feature information from different resolutions while maintaining high-resolution feature maps, which enables it to better capture subtle structural changes and tumor characteristics, especially for complex medical imaging tasks. Compared with traditional convolutional neural networks (CNNs), HRNet can effectively maintain spatial detail information in images and reduce the loss of fine features in low-resolution layers, thereby improving the accuracy of staging prediction. Its multi-scale feature fusion mechanism enables the model to extract useful information at different scales, enhance the ability to recognize subtle changes in ovarian cancer, and especially has obvious advantages in recognizing key parts such as tumor edges and lesion areas. Second, from the perspective of task requirements, the diagnostic task of ovarian cancer staging usually requires identifying and distinguishing subtle differences in tumors, as well as evaluating the spread of tumors. This requires the model to capture high-dimensional spatial features in images, such as tumor shape, density, and location. HRNet ‘s architecture is particularly suitable for handling this complex image classification task. It can provide more accurate classification results while maintaining high resolution and combining depth information. For ovarian cancer staging, the distribution and morphological differences of the tumor are important bases for staging, and HRNet can effectively capture these differences, thus improving the staging accuracy of the model.

The main clinical significance of this study is that it provides an efficient and objective decision-making tool for preoperative staging of cervical cancer, especially in distinguishing between IA–IB2, IIA1 and IB3, IIA2–IVB. In the current guidelines, surgical treatment is usually recommended for patients with stage I cervical cancer, while patients with IB3, IIA2–IVB are more suitable for concurrent chemoradiotherapy. Therefore, accurate preoperative staging is crucial for the selection of treatment options. However, traditional staging methods mainly rely on the experience and judgment of radiologists, which are greatly affected by subjective factors, and for inexperienced doctors, it may be difficult to distinguish the tumor characteristics of IA–IB2, IIA1 and IB3, IIA2–IVB. The deep learning staging model constructed by HRNet can provide objective staging basis with high accuracy (69.70%) and discrimination ability (AUC value 0.7778). The model can capture key features in MRI images that are closely related to tumor staging, such as the clarity of tumor boundaries, the contrast between tumors and surrounding tissues, and possible local infiltration information, thus playing an important role in the difference in imaging performance between IA–IB2, IIA1 and IB3, IIA2–IVB. For preoperative decisions recommended by the guidelines, the application of this model can reduce the misjudgment of staging due to human subjective factors, thereby improving the accuracy and efficiency of clinical decision-making. In addition, the automated staging tool developed in this study can significantly save clinical resources. In cases where primary medical institutions or imaging doctors are inexperienced, the AI-assisted system can provide a reference for decision-making and reduce the risk of overtreatment or delayed treatment.

Although this study demonstrated the excellent performance of HRNet in preoperative staging of cervical cancer, there are still some limitations. First, the amount of data in this study is relatively limited, including only MRI imaging data of 112 patients, which may affect the generalization ability of the model. Especially when multi-center data are involved, the heterogeneity of different scanning devices, imaging parameters, and patient groups may affect the applicability of the model. Second, this study did not verify the external independent data set, and the robustness of the model has not been fully evaluated. In the future, multi-center studies are needed to further verify the generalizability of the model. In addition, the current task of the model focuses on the binary classification of IA–IB2, IIA1 and IB3, IIA2–IVB, while the staging of cervical cancer is more complicated in actual clinical practice. In the future, it is possible to consider expanding the model to cover more staging categories to meet a wider range of clinical needs. Finally, the model of this study is still in the laboratory stage and has not yet been integrated into the clinical workflow. Its efficiency, interactivity, and acceptability in the actual clinical environment need to be further explored. These limitations provide improvement directions for future research, including the expansion of data scale, multi-center verification, and optimization and integration of models in actual clinical applications.

## 5 Conclusion

This study developed a deep learning-based preoperative staging prediction model for cervical cancer. By training and verifying multiple CNNs on MRI images of 112 patients, the HRNet model showed the best performance in the binary classification task of surgery-recommended group (IA–IB2, IIA1) and the group recommended for concurrent chemoradiotherapy or systemic treatment (IB3, IIA2–IVB), with an accuracy of 69.70% and an AUC value of 0.7778. Studies have shown that the model can effectively capture the key imaging features of tumors and provide an objective and efficient auxiliary tool for preoperative treatment decisions (surgery or synchronous chemoradiotherapy). Compared with traditional image analysis methods, this model significantly reduces the impact of human subjectivity on staging results and improves the accuracy and efficiency of staging. In the future, this technology can be further improved through multi-center data expansion, model optimization and clinical integration.

## Data Availability

The raw data supporting the conclusions of this article will be made available by the authors, without undue reservation.
